# COVID-19 and Cancer Care: A Review and Practical Guide to Caring for Cancer Patients in the Era of COVID-19

**DOI:** 10.3390/curroncol31090393

**Published:** 2024-09-10

**Authors:** Simon Claveau, Farhan Mahmood, Baraa Amir, Jennifer Jing Wah Kwan, Cheryl White, Joe Vipond, Lisa Iannattone

**Affiliations:** 1 Department of Medicine, McGill University, Montreal, QC H3A 0G4, Canada; 2 College of Medicine, Imam Abdulrahman Bin Faisal University, Dammam 31441, Saudi Arabia; 3 Independent Researcher, Burlington, ON L7N 3V2, Canada; jenniferjwkwan@gmail.com; 4 Independent Researcher, Toronto, ON M6P 3X9, Canada; 5 Department of Emergency Medicine, University of Calgary, Calgary, AB T2N 1N4, Canada

**Keywords:** COVID-19, SARS-CoV-2, infection prevention, cancer care

## Abstract

COVID-19, a novel infectious disease caused by the emergence of the SARS-CoV-2 virus in 2020, has had a profound impact on healthcare, both at the individual and population level. The impact at the population level was felt most acutely during the emergency phase of the pandemic, with hospital capacity issues leading to widespread disruptions and delays in the delivery of healthcare services such as screening programs and elective surgeries. While hospitals are no longer being acutely overwhelmed by COVID-19 patients, the impact of the virus on vulnerable patient populations such as cancer patients continues to be of ongoing consequence. Cancer patients remain at high risk of hospitalization, ICU admission, and death due to COVID-19, even in the era of vaccination. Infection prevention and risk mitigation strategies such air quality control, masking, testing, vaccination, and treatment should therefore be integrated into the usual care and counseling of cancer patients moving forward to avoid preventable morbidity and mortality from this infection and ensure the safety of this vulnerable cohort as they navigate their cancer diagnosis and treatment in the era of COVID-19.

## 1. Introduction

COVID-19, caused by the SARS-CoV-2 virus, has had a profound impact on global health since its emergence in 2019. The pandemic has not only overwhelmed healthcare systems but has also posed severe risks to vulnerable populations, including patients with cancer. GLOBOCAN estimates that close to 20 million people were diagnosed with cancer in 2022 and that approximately one in five people will develop cancer in their lifetime. These individuals are particularly susceptible to COVID-19 due to immune system compromise from the malignancy itself, side effects of anti-cancer treatments, and increased contact with the healthcare system, which may result in greater exposure to the SARS-CoV-2 virus. The interplay between COVID-19 and cancer care is multi-faceted and complex. This review aims to provide a broad overview of the existing literature on this topic, highlighting the impact of the COVID-19 pandemic on cancer care, the clinical outcomes of COVID-19 in cancer patients, and the prevention and therapeutic strategies that can be used to mitigate COVID-19-related risks for these patients, with a focus on North American guidelines. This practical guide will help inform clinical practice and emphasize the need for ongoing vigilance and patient counseling with regards to the SARS-CoV-2 virus.

## 2. The Impact of the COVID-19 Pandemic on Cancer Care

**Figure 1:**
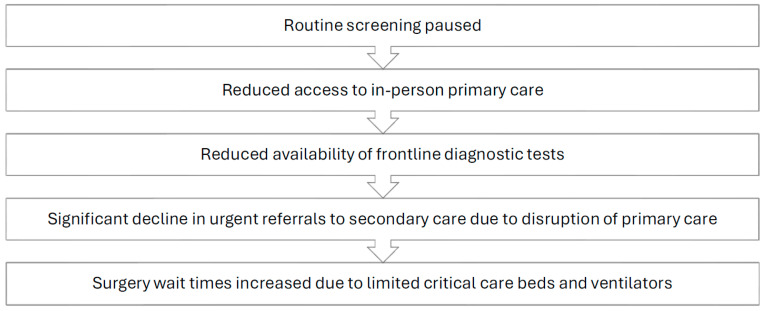
Impact of the COVID-19 pandemic on the oncology care pathway.

The screening and workup of malignancies is a time-sensitive matter that requires prompt implementation of guideline-oriented protocols by competent staff who work in a well-functioning healthcare system. Unfortunately, the COVID-19 pandemic resulted in significant disruptions to the standard pathway commonly followed by patients seeking cancer screening, as well as patients with existing malignancies requiring treatment (Figure 1). Proficient cancer care is a result of a dynamic interplay between three key entities: screening protocols, healthcare workers, and the healthcare system. The aforementioned key players were all impacted by the global pandemic to varying degrees.

The COVID-19 pandemic greatly impacted the lives of patients suffering from various medical and surgical conditions, with oncology patients being a particularly affected group [1,2]. The large-scale lockdowns that occurred in many countries, in addition to the heightened level of fear due to a rapidly spreading novel pathogen at the time, led to an increased reluctance among individuals to visit their primary care practitioners for routine testing and screening [3,4]. A survey conducted through the Council of Academic Family Medicine’s Educational Research Alliance, an American group, found that up to 34.5% of respondents reported postponing cancer screening, with many physicians reporting that patients were afraid to come into the office [5,6]. Additionally, several medical governing bodies, including the European Society for Medical Oncology and the American Cancer Society, recommended that at-risk patients cease routine screening protocols during the peak of the pandemic. This, combined with the aforementioned factors, led to a seismic decrease in the number of screened patients for commonly prevalent cancers [7,8,9]. In the United States (US), between the months of January and April 2020, breast, colon, and cervical cancer screening rates dropped by a massive 94%, 86%, and 94%, respectively [10]. The province of Ontario, Canada showed similar findings, with a 41% reduction in screening rates in 2020 compared to the prior year [11]. This observable decrease in screening across several jurisdictions and various countries posed a massive problem to at-risk groups, as their malignancies would go undetected during the peak of the COVID-19 pandemic. The interruption of breast cancer screening, even for a period of only 3 months, may lead to a 7% decrease in total diagnosed cases per year, with a 6-month interruption potentially leading to a 14% decrease [12]. A national survey conducted in the United States estimated that over 134,000 cancer cases may have gone undiagnosed between March 2020 and December 2020 [13]. According to the National Cancer Institute in the US, the significant screening delays and cancellations during the spring of 2020 are expected to result in nearly 10,000 excess deaths from breast and colorectal cancer alone within the next decade [14].

In addition to the disruption of screening programs during the pandemic, access to general practitioners and primary care physicians was restricted in an effort to control the outbreak of SARS-CoV-2. As a result, referrals dropped significantly, with institutions such as the National Health Services (NHS) in the UK observing a 60% reduction in referrals to secondary care centers [15]. Furthermore, the difficult working conditions associated with the crisis increased the prevalence of physician burnout, depression, and overall stress [16]. In a survey of 206 large healthcare organizations in the US, 50% of healthcare workers met the criteria for burnout, with nurses being the most affected group at 56%. Nurses were also the group most likely to report their intent to leave at 41% [17]. These alarming figures illustrate the tremendous burden carried by key actors of the healthcare system since 2020. The psychological distress experienced by healthcare workers on the front lines was multifactorial in etiology, with prominent causes including significant personal protective equipment (PPE) shortages, increased documentation requirements, fear of a newly spreading disease, and high patient volumes, which, combined with previously described disruptions and delays, contributed to reduced healthcare service quality and moral injury [14,18].

The SARS-CoV-2 virus itself also contributed to the negative toll of the pandemic on the healthcare workforce. A study by the Institut national de la santé du Quebec (INSPQ) that was presented at the 1st Canadian Symposium on Long Covid found that 10% of all healthcare workers suffered from the effects of long COVID lasting more than 12 weeks, with a third experiencing severe symptoms. More than half of the healthcare workers in the study had been experiencing long COVID symptoms, such as shortness of breath, fatigue, memory loss, and confusion for longer than a year. The majority developed long COVID after January 2022, subsequent to an omicron infection or reinfection. Seventy percent reported that their symptoms had an impact on their work [19].

The staffing issues that have plagued hospitals since the start of the pandemic, with a survey of American healthcare workers revealing that 18% had left their jobs during the pandemic [20], in addition to the high volume of COVID-19 patients that required critical care beds and ventilators, resulted in significantly increased wait times for diagnostic tests and elective surgeries, including cancer surgeries [15]. It is estimated, based on global surgical data, that over a 12-week period of peak disruption during the pandemic, 37.7% of cancer surgeries were postponed or canceled [21]. These disruptions in cancer care, which have occurred at multiple points in the patient care pathway and on more than one occasion since the start of the pandemic, can be expected to result in an aftershock period where physicians and healthcare systems must contend with an increased burden of more advanced stage cancers due to disease progression from delays in care and from undetected malignancies eventually presenting at more advanced stages. It is also hypothesized that some patients may experience rapid disease progression after COVID-19 infection due to immune dysregulation [22,23], which could potentially further add to the burden of advanced staged disease.

## 3. COVID-19 Outcomes in Cancer Patients

Patients living with cancer are very often immunocompromised because of their disease and/or their anti-cancer treatments. They are also often older than 60 years old and have other co-morbidities in addition to their cancer diagnosis and treatment. These compounding risk factors make this patient population highly vulnerable to COVID-19 infections. It is thus of vital importance for both cancer patients and their physicians to be aware of this new risk as these patients will require enhanced preventive measures, surveillance, and counseling going forward.

A systematic review and meta-analysis published in the first year of the pandemic found that the rate of severe or critical disease in cancer patients with COVID-19 was 45.5% (odds ratio (OR) = 3.91), the rate of admission to the intensive care unit (ICU) was 14.5% (OR = 3.10), the rate of mechanical ventilation was 11.7% (OR = 4.86), and the mortality rate was 21.1% (OR = 3.23) when compared to non-cancer patients [24]. Similar findings have been reported in other large-scale systematic reviews and meta-analyses, as well [25,26]. This increased risk of severe COVID-19 outcomes was echoed by a prospective study highlighting a higher risk of severe events (ICU admission, mechanical ventilation, and death) among patients with cancer compared to those without cancer, at 39% versus 8%, respectively [27]; the increased risk persisted after adjusting for age and smoking status [28], although one systematic review found that adjusting for age consistently resulted in lower estimated ORs of COVID-19-related death in cancer patients (adjusted OR = 1.37) [29]. Interestingly, COVID-19-related mortality may decrease with time since initial diagnosis and treatment of cancer (adjusted OR 1.55 versus 0.98 at 1 year versus 5 years since cancer diagnosis/treatment, respectively) [30]. Finally, hematological malignancies have been shown to have a higher mortality risk when compared to solid tumors (OR = 1.86) [31].

The clinical outcomes of cancer patients with COVID-19 may also be influenced by the type of treatment they are receiving. Large studies have found that patients with hematologic malignancies undergoing chemotherapy treatment and patients receiving surgical treatments for their cancer had a higher risk of clinically severe events compared to patients who were not receiving these treatments. No significant differences were found in COVID-19 outcomes in patients receiving cancer therapies for solid tumors [27,32]. Similarly, evidence suggests that there is no increase in mortality or ICU/hospitalization rates in cancer patients receiving immune checkpoint inhibitors compared to cancer patients who are not [33,34,35]. COVID-19 infection in the context of hematopoietic cell transplantation (HCT), on the other hand, has been shown to be associated with increased mortality, mechanical ventilation, and ICU admission. Higher death rates were found in patients who developed COVID-19 within 12 months of HCT (risk ratio (RR) = 2.11), within 6 months of receiving immunosuppressant drugs (RR = 2.11), and in the context of graft vs. host disease (RR = 2.38) [36].

COVID-19 infections may also negatively impact cancer treatment in other ways. For example, SARS-CoV-2-positive cancer patients have been shown to be significantly more likely to experience hematotoxicity to their anti-cancer treatments when compared to patients that did not test positive for SARS-CoV-2 (73% vs. 35%), leading to more treatment delays in the patients that contracted COVID-19 [37]. As with the general population, COVID-19 infections in cancer patients also carry the risk of developing long-COVID. The rate of long-COVID in this patient population, defined as the persistence/worsening of symptoms or one or more sequalae following acute COVID-19 infection, varies considerably across studies. Long-COVID has been reported to occur in 51.3% (*n* = 80), 16.6% (*n* = 186), 12.4% (*n* = 97), 60% (*n* = 312), and 8% *(n* = 186) of cancer patients at 4 weeks, 2.3 months, 12 weeks, 7 months, and 12 months after the acute COVID-19 diagnosis, respectively [38,39,40,41]. It should be noted, however, that these studies lacked control groups, and many symptoms of long-COVID overlap with known side effects of anti-cancer therapies and cancer itself. More research is needed in this area to quantify the risk of long-COVID in this patient population.

Many of the data available on the risks associated with COVID infections in cancer patients stems from research conducted before the vaccine rollout; however, it is important to note that vaccination benefit may be reduced in this patient population as many cancer patients, such as those with hematologic malignancies and HCT recipients, experience reduced immunogenicity to COVID-19 vaccines [42,43,44,45]. Nevertheless, vaccination has been shown to significantly reduce the risk of poor COVID-19 outcomes in this vulnerable population. An observational cohort study comparing unvaccinated cancer patients to those that had been vaccinated found that the incidence of hospitalization was 42% vs. 29%, respectively; the incidence of mechanical ventilation was 8.4% vs. 4.6%; and all-cause mortality within 30 days of COVID-19 diagnosis was 17% vs. 4.65% [46]. In a case–control study of vaccinated patients with hematologic malignancies, those who had received a booster vaccine dose had reduced odds of severe disease (aOR = 0.73); however, the proportion of patients that experienced severe COVID-19 (21.3%) or died within 28 days of a positive SARS-CoV-2 test result (3.5%) remained high in this highly vaccinated cohort, suggesting that these patients remain extremely vulnerable to COVID-19 even in the post-vaccination era [47].

## 4. Treatment of COVID-19 Infections in Cancer Patients

Given the risks associated with COVID-19 in cancer patients, if infection should occur, treatment options to reduce the risk of a negative outcome should be carefully considered. As we previously described, it is well-documented that cancer, either from the pathology itself or its treatment, can increase the risk of progression to severe COVID-19 disease and all-cause mortality [25,48]. Since the start of the pandemic, multiple treatments have been studied, some with beneficial results and others with disappointing outcomes. In this section, the available evidence on COVID-19 treatments will be reviewed to provide insight on how to treat cancer patients with confirmed COVID-19 infections.

Upon initial evaluation, it is crucial to determine where the patient lies on the clinical spectrum of disease (i.e., asymptomatic, pre-symptomatic, mild, moderate, severe, or critical). In addition, whether the patient is hospitalized or requires supplemental oxygen will also influence the therapeutic algorithm [48]. As a guiding principle, since cancer patients are considered a high-risk group, they are eligible to receive anti-COVID-19 medication in the outpatient setting for mild-to-moderate disease. COVID-19 treatments can be divided into several categories, including antiviral agents, anti-SARS-CoV-2 monoclonal antibodies, immunomodulatory agents, antithrombotic therapies, and miscellaneous drugs. While a comprehensive review of all COVID-19 therapies reported in the literature is beyond the scope of this review, this section will focus on the therapeutics that have been most studied or used to date. Table 1, adapted from the National Institutes of Health (NIH) COVID-19 guidelines, summarizes the recommendations and level of evidence for each of these treatment options [48]. These guidelines were last updated in February 2024 and come from the consensus of numerous American-based federal agencies and professional societies. The level of evidence “A” designates a strong recommendation, “B” for moderate, and “C” for weak.

Nirmatrelvir/Ritonavir (Paxlovid^MC^) is an anti-viral agent approved by Health Canada and strongly recommended by the World Health Organization (WHO) for the treatment of patients with mild-to-moderate COVID-19 infection at high risk of progression to severe disease [49]. The approval of this agent was based on the EPIC-HR phase 2/3 trial, which enrolled nonhospitalized adults with mild-to-moderate disease who were not vaccinated and at high risk of progressing to severe disease. It demonstrated an 89% relative risk reduction in COVID-associated hospitalization or death with twice-daily treatment for five days, when initiated within five days of symptoms onset [50]. However, it is worth mentioning that ritonavir is a potent P450 3A4 inhibitor, which may cause drug–drug interactions with patients’ other medications, including their anti-cancer medications [51]. If in doubt, it is recommended to consult a pharmacist for assistance.

The second anti-viral agent commonly used to treat COVID-19 is Remdesivir. It is FDA- and Health Canada-approved for the treatment of COVID infection in nonhospitalized patients with mild-to-moderate disease who are at high risk of progression to severe disease [49]. It should be started within seven days of symptom onset and administered for three days [48]. If the patient is hospitalized, the treatment should be continued for five days or until the end of their hospital stay. Multiple clinical trials were conducted to provide these recommendations, including the PINETREE trial in nonhospitalized patients, which showed that three consecutive days of Remdesivir resulted in an 87% relative reduction in the risk of hospitalization or death when compared to that in a placebo group [52]. In hospitalized patients, the ACTT-1 trial demonstrated that the time to recovery was reduced in patients with severe COVID [53]. The benefits were greater when treatment was initiated within 10 days of symptom onset and in patients receiving supplemental oxygen. Its intravenous formulation makes Remdesivir less convenient for the outpatient setting, unfortunately; however, multiple provinces in Canada have organized community infusion centers that enable delivery of this medication in the outpatient setting without the need for hospitalization [53,54].

The next category includes monoclonal antibodies against SARS-CoV-2 spike proteins. Many of these products received emergency FDA approval during the early pandemic period for the treatment of mild-to-moderate disease, including bamlanivimab plus etesevimab, casirivimab plus imdevimab, sotrovimab, and bebtelovimab [55,56,57,58]. However, none of these are currently FDA-approved because they were judged to be non-efficacious for the later COVID variants and subvariants such as Omicron [48].

Another broad class of COVID treatments includes immunomodulators such as systemic corticosteroids. There are strong data for the use of corticosteroids from several trials that demonstrated improved clinical outcomes and reduced mortality in hospitalized COVID patients requiring supplemental oxygen [59,60]. The underlying mechanism is thought to be decreased systemic inflammation which, when left unabated, can lead to lung injury and multiorgan dysfunction in severe disease. The landmark trial, RECOVERY, showed reduced mortality in the cohort receiving 6 mg dexamethasone for 10 days plus the standard of care compared to those receiving the standard of care alone. However, there are no available data to support its use in nonhospitalized patients not requiring supplemental oxygen [61,62]. Other systemic corticosteroids such as methylprednisone and hydrocortisone have been studied, but evidence for their use is limited due to small sample sizes. Therefore, these should only be used in situations where dexamethasone is not readily available [48]. Additional immunomodulators that can be used to treat COVID-19 are tocilizumab (interleukin-6 inhibitor), baricitinib (janus kinase inhibitor), abatacept, and infliximab. Combining their use with systemic steroids is recommended for patients with severe-to-critical disease exhibiting increased oxygen requirements or systemic inflammation despite already being on dexamethasone [48]. In this scenario, baricitinib or tocilizumab are preferred [63,64].

The final category of COVID-19 treatments for discussion includes various miscellaneous drugs studied in randomized trials. This includes fluvoxamine, intravenous immunoglobulins (IVIG), ivermectin, and metformin. Fluvoxamine, a selective serotonin reuptake inhibitor, was studied in at least six clinical trials [48]; however, no significant benefit was found for preventing hospitalization or death either in vaccinated or unvaccinated patients [65,66]. A similar scenario also applies for IVIG. Studies showed uneven levels of neutralizing activity against SARS-CoV-2 variants which was likely related to which variant was dominant at the time of plasma collection [67]. The inconsistent results render the data too weak to support its use [48]. As for ivermectin, which is FDA-approved as an antiparasitic medication, at least four randomized trials compared it against a placebo in the outpatient setting. However, all trials failed to demonstrate clinical benefits in terms of progression to severe disease, hospitalization, or death. It is consequently not recommended in the treatment of COVID [48]. The last agent is metformin, identified because of its antiviral, anti-inflammatory, and antithrombotic properties [68]. The TOGETHER and COVID-OUT trials assessed its efficacy for nonhospitalized patients [65,69] and found that it did not reduce the risk of hospitalization or death. Consequently, the data are insufficient to support its use [48].

## 5. COVID-19 Infection Prevention Strategies

As discussed in previous sections, the evidence overwhelmingly demonstrates that cancer patients remain vulnerable to severe outcomes of COVID-19 infections, even in the era of vaccination. While there are treatment options that may mitigate the risk of negative outcomes as discussed above, the benefit and accessibility of these treatments is limited. Infection prevention strategies are thus essential for oncology patients. It is incumbent for physicians and healthcare workers to ensure the safety of these patients in the healthcare setting, while offering counseling and education on strategies to protect themselves from COVID-19 in the community. The following section will review current recommendations and evidence regarding indoor air quality, masking, rapid antigen testing, and vaccination guidelines.

Although the World Health Organization (WHO) recommended that COVID-19 no longer fits the definition of a Public Health Emergency of International Concern in May 2023, the organization has continued to emphasize that the pandemic is not over. As recently as February 2024, Maria Van Kerkhove, interim director of the WHO’s Department of Epidemic and Pandemic Preparedness and Prevention, has stated publicly that we are still in a pandemic and that COVID-19 is still a global health risk, and she expressed concern at the complacency seen at the government level in many countries [70]. This complacency is of notable concern for the safety of high-risk groups such as oncology patients, particularly in healthcare settings, given that these patients are in frequent contact with the healthcare system.

In April 2024, the WHO released a technical report recognizing that COVID-19 is predominantly spread through the air by airborne inhalation/transmission, with direct deposition (formerly known as droplet transmission) representing a much smaller risk [71]. Recognizing that the mode of transmission of COVID-19 is primarily through airborne inhalation is central to providing high-impact practical guidance to patients and healthcare providers to limit the spread of COVID-19 in the community and in healthcare settings.

The Public Health Agency of Canada (PHAC) recommends applying a hierarchy of controls to prevent the spread of respiratory infectious diseases in healthcare settings [72]. The hierarchy of controls, which is similar to the “Swiss cheese” model of infection prevention, utilizes multiple, layered interventions to prevent the spread of infection. In the case of respiratory pathogens like SARS-CoV-2, the hierarchy of controls involves administrative controls, engineering controls, and personal protective equipment. Administrative controls are policies and procedures that are intended to decrease the chances that the virus is present in an establishment. Examples of administrative controls include policies that encourage workers to stay home when ill and facilitating access to testing to detect and remove cases of infection from the environment, as well as infrastructure that allows patients greater access to high-quality telemedicine where possible [72].

Since asymptomatic transmission accounts for more than 50% of COVID-19 infections, symptom-based administrative controls are insufficient in isolation [73,74]. The risk of asymptomatic transmission in healthcare facilities can be mitigated with testing and universal masking policies which will be further discussed below. Rapid antigen tests (RAT) provide another tool that can allow oncology patients to minimize the risk of being inadvertently exposed to the virus by those they interact with in the community. While the sensitivity of a single RAT is 83% in symptomatic individuals and only 39% in those without symptoms, the sensitivity of these tests was significantly improved with repeat testing in a recent study. In this study, testing twice, 48 h apart, increased the sensitivity of RAT to 93% in symptomatic participants, while testing three times increased the sensitivity to 79% in asymptomatic participants [75]. It should be noted that a positive RAT result is correlated with infectivity, and therefore, patients should avoid contact with positive individuals where possible until their RAT is negative on two separate occasions, at least 24 h apart [76,77].

Occasionally, oncology patients may find themselves unable to avoid contact with positive contacts, such as when someone in their home has COVID-19. In this instance, patients can be referred to patient-friendly online resources such as the *Clean Air Crew* website that details how to isolate from an infectious occupant inside a home or apartment [78].

The next broad category in the hierarchy of controls, or Swiss cheese model, is engineering controls [72]. In the healthcare setting, engineering controls include optimizing indoor air ventilation and filtration to reduce the viral load in the air should an infectious person be present in the establishment. While an in-depth overview of indoor air engineering controls is beyond the scope of this review, Public Health Ontario has published a detailed guide on this topic, Heating, Ventilation and Air Conditioning (HVAC) Systems in Buildings and COVID-19, that notes that improper or insufficient ventilation has been frequently reported as a risk factor in outbreak investigations [79]. Patients should also be encouraged to improve the indoor ventilation of their homes if possible, especially if an infectious person is in the house or if there are high levels of community spread, which can be assessed using public wastewater monitoring data and resources such as COVID-19 Resources Canada’s biweekly COVID forecast [80]. The Public Health Agency of Canada (PHAC) has created patient-friendly resources on this topic that can be found on the COVID-19: Improving indoor ventilation page [81]. This page includes a video on ways to improve ventilation and air filtration in the home, as well as a printable guide on choosing the best air purifier for your home, among other resources.

The final category in the hierarchy of controls is masking [72]. It is important to note that masks, both in the healthcare setting and the community, serve dual purposes: source control and personal protective equipment (PPE). Source control means lowering the risk that an infected person infects others. PPE means lowering the risk that an uninfected person gets infected. When using masks as PPE to control the hazard at the individual level, they are intended to limit the inhalation of infectious particles. While masking is no longer mandated by law, organizations such as the National Cancer Institute and PHAC continue to stress the importance of wearing a mask in the community setting for those at increased risk of more severe disease or outcomes, when around others who are at high risk of more-severe disease or outcomes, when visiting a group living setting, and/or when in a crowded or poorly ventilated setting [81]. In the community setting, the PHAC recommends choosing the best quality and best-fitting mask, which essentially means a respirator mask such as an N95, KN95, or elastomeric mask. PHAC notes that respirators do not require formal fit testing for use in the community [82]. Masks are considered well-fitted when there are minimal gaps on the edges, including around the nose and sides, to ensure air is properly filtered through the mask. Cloth masks and non-medical masks provide limited and inconsistent protection, and patients and their caregivers should therefore be encouraged to use well-fitting medical-grade masks or respirators [82].

The other usefulness of masks in the hierarchy of controls is as source control to limit the spread of the wearer’s exhaled respiratory particles. Much like masks in the community setting, masks used for the purpose of source control are not required to be fit-tested, but they should be as well-constructed and well-fitting as possible, as noted by groups such as the Canadian Center for Occupational Health and Safety (CCOHS) [83]. While universal masking has been discontinued outside of healthcare contexts for some time, there is ongoing debate about the ethics of discontinuing masking in healthcare facilities, with several patient advocates and advocacy groups calling for continued masking in light of the fact that COVID-19 is here to stay and is now in circulation year-round [84]. These calls for vigilance are notable in the oncology milieu given the high risk of severe outcomes previously described in this article. It is also important to note that for many, attending healthcare spaces is not a choice but is required for active illness management. One advocate, Christine Mitchell, a public health researcher and caregiver to her father who is battling colorectal cancer, remarked that “It’s very jarring to have so much fear about going to a place that I am going to protect my health or my father’s going to get treatment, and being fearful that it’s actually endangering our health” [85].

The reasons cited for reverting to masking only in certain circumstances in healthcare settings are often similar to those given for community settings. It is felt that since immunity acquired through vaccination and infection has greatly reduced the morbidity and mortality of COVID-19, the risk is now similar to that of other respiratory infections, and these risks have long been tolerated. A perspective piece in the New England Journal of Medicine noted that this framing has limited application in healthcare settings for two main reasons. The first is that hospitalized patients, or in the case of this review, oncology patients, are different from nonhospitalized patients, or non-cancer patients. Hospitals and healthcare facilities, by definition, aggregate subgroups of the population that continue to be at elevated risk for severe disease and death. The second reason is that nosocomial infections caused by viruses other than SARS-CoV-2 are common, underappreciated, and are also associated with adverse health effects in vulnerable populations [86]. These include influenza, RSV, or other infections that may delay or negatively affect treatment outcomes. Healthcare workers should therefore seek to reduce the risk of nosocomial transmission of all respiratory pathogens to vulnerable patients. The authors note that viewed through this lens, continued masking in healthcare settings makes sense, especially given that viral illnesses can be spread by staff and visitors with mild or asymptomatic infections. The authors go on to acknowledge that healthcare workers may be experiencing masking fatigue and that therefore masking requirements can be tied to levels of viral transmission and the individual patients’ risk of severe disease. When applying these recommendations to the care of cancer patients, a patient population known to be at increased risk of severe disease and death, universal masking for the protection of these patients seems to be a prudent and justified course of action. Indeed, the Centers for Disease Control and Prevention (CDC) and PHAC both continue to recommend that healthcare facilities consider broad masking for source control in certain clinical contexts, such as when working with immunocompromised persons or those at greater risk of acquiring an infection [87,88].

Unfortunately, oncology patients may acquire COVID-19 despite the prevention measures discussed as part of the hierarchy of controls. Up-to-date vaccination therefore remains an essential harm-reduction tool in the care of oncology patients. Beginning in the fall of 2024, the National Advisory Committee on Immunization (NACI) has recommended the most recently updated COVID-19 vaccination for previously vaccinated and unvaccinated individuals at increased risk of SARS-CoV-2 infection or severe COVID-19 disease such as those with cancer (among other groups) [89]. For previously unvaccinated individuals who are moderately-to-severely immunocompromised, the NACI recommends that patients receive three doses. New recipients of hematopoietic stem cell transplantation (HSCT) or chimeric antigen receptor (CAR) T-cell therapy are considered immunologically naïve and the NACI recommends that they be vaccinated with three doses beginning at 3 to 6 months post-HSCT/CAR T-cell therapy, regardless of vaccination or infection history prior to transplant/therapy [88]. Finally, previously vaccinated individuals that are moderately-to-severely immunocompromised are most often eligible for additional booster COVID-19 vaccine doses after 3–6 months. In the spring of 2024, NACI guidelines allowed for this patient population to receive a first dose of the updated XBB.1.5 vaccine in the fall of 2023 and a second dose in the spring of 2024, with a recommended interval of 6 months [90]. Similarly, the CDC’s current guidelines state that immunocompromised individuals under the age of 65 may receive an additional (second) dose of any updated vaccine, and immunocompromised individuals over the age of 65 should receive an additional (second) dose of any updated vaccine at least 2 months after the last updated vaccine dose [91]. It is important for clinicians to review these guidelines periodically to ensure that oncology patients are properly counseled on the most recent COVID-19 vaccination eligibility guidelines and recommendations.

## 6. Conclusions

Since the introduction of the SARS-CoV-2 virus into circulation in 2020, cancer patients have faced new risks and unique challenges related to their health status and the ongoing circulation of this novel pathogen. The COVID-19 pandemic compromised access to healthcare services, disrupting screening programs, negatively affecting surgical wait times, and introducing a new nosocomial and community infection risk that oncology patients and healthcare teams must contend with. Numerous studies have shown that cancer patients are at increased risk of severe outcomes and death in the event of a COVID-19 infection, even after vaccination. The risk is therefore ongoing and ever-present for these patients. Fortunately, there is quality evidence to support the use of multi-pronged disease prevention measures, both at the individual level and through broader public health strategies. These include air quality engineering controls, testing and other administrative controls, masking, and vaccination. Additionally, with the assistance of their care teams, patients can also access antiviral therapies in the outpatient setting that may limit progression to severe COVID-19 disease and decrease mortality should they contract the virus. As the situation is still evolving, and new vaccines and treatments are expected to emerge in the future, continuous monitoring of new tools and evidence and adherence to updated recommendations and guidelines are essential components of modern-day cancer care that can mitigate risk and ensure optimal outcomes for this vulnerable patient population in the era of COVID-19.

## Figures and Tables

**Table 1:** Summary of recommendations for anti-COVID therapies in cancer patients.

Therapy	Setting	Recommendation	Evidence
Nirmatrelvir/Ritonavir (Paxlovid)	Nonhospitalized, mild to moderate disease	IN FAVOR, start within 5 days of symptoms onset	A
Remdesivir	Nonhospitalized and hospitalized, mild to severe disease	IN FAVOR, start within 7 days of symptoms onset	B
Monoclonal antibodies	Nonhospitalized, mild to moderate disease	AGAINST	B
Dexamethasone	Nonhospitalized	AGAINST	A
	Hospitalized, requiring oxygen	IN FAVOR	B
Baricitinib	Hospitalized, requiring high-flow oxygen	IN FAVOR, preferred option	B
Tocilizumab	Hospitalized, requiring high-flow oxygen	IN FAVOR, preferred alternative	B
Abatacept	Hospitalized, requiring high-flow oxygen	IN FAVOR, additional alternatives	C
Infliximab	Hospitalized, requiring high-flow oxygen	IN FAVOR, additional alternatives	C
Fluvoxamine	Nonhospitalized, mild to moderate disease	AGAINST	A
Intravenous immunoglobulins	Nonhospitalized, mild to moderate disease	AGAINST	A
Ivermectin	Nonhospitalized, mild to moderate disease	AGAINST	A
Metformin	Nonhospitalized, mild to moderate disease	AGAINST	B
